# Directional Auxetic Behavior of Mechanical Metamaterials: Material-Dependent and Geometry-Driven Mechanisms

**DOI:** 10.3390/ma18225103

**Published:** 2025-11-10

**Authors:** Barbara Schürger, Jozef Bocko, Peter Frankovský, Ingrid Delyová, Ján Kostka

**Affiliations:** Department of Applied Mechanics and Mechanical Engineering, Faculty of Mechanical Engineering, Technical University of Košice, Letná 1/9, 042 00 Košice, Slovakia; jozef.bocko@tuke.sk (J.B.); peter.frankovsky@tuke.sk (P.F.); ingrid.delyova@tuke.sk (I.D.); jan.kostka@tuke.sk (J.K.)

**Keywords:** auxetic metamaterials, negative Poisson’s ratio, finite element analysis (FEA), re-entrant lattices, chiral and anti-chiral structures, material–geometry interaction, anisotropy in mechanical behavior, structural design applications

## Abstract

Mechanical metamaterials derive their unconventional properties from geometry rather than composition, enabling phenomena such as negative Poisson’s ratio and tunable stiffness. This study presents a systematic finite element analysis of three canonical auxetic topologies—re-entrant, chiral, and anti-chiral lattices—subjected to uniaxial loading in two orthogonal directions. Four engineering metals (steel, copper, aluminum, titanium) were analyzed to evaluate how material stiffness interacts with geometry in defining auxetic response. A detailed mesh-convergence and sensitivity analysis ensured numerical reliability and isolation of geometric effects within the linear-elastic regime. The results reveal three distinct mechanisms: (i) material-sensitive auxeticity in re-entrant lattices, which achieved the most extreme negative Poisson’s ratios (ν < −2.0) but with strong dependence on stiffness; (ii) directional auxeticity in chiral lattices, which exhibited negligible response under X-loading but significant negative values (ν ≈ −0.5) under Z-loading; and (iii) geometry-dominated auxeticity in anti-chiral lattices, which remained robust and nearly material-independent (ν ≈ −1.2). This comparative framework clarifies the balance between geometry- and material-driven mechanisms, extending prior single-material or single-geometry studies. The findings provide design guidelines for selecting auxetic topologies depending on whether robustness, tunability, or maximum auxetic effect is required, with direct implications for protective equipment, aerospace structures, and biomedical scaffolds.

## 1. Introduction

Mechanical metamaterials are artificially engineered architectures designed to exhibit effective properties beyond those of natural materials. In recent decades, research on metamaterials has expanded rapidly across disciplines, including optics, acoustics, and mechanics. Mechanical metamaterials are of particular interest because of their unconventional responses—such as negative compressibility, programmable stiffness, and auxeticity—originate from architecture and topology rather than chemical composition [1,2].

The term auxetic originates from the Greek word auxetos, meaning “that which grows.” The first experimental demonstration of materials with a negative Poisson’s ratio was reported by Lakes [3] while the term auxetic was later popularized to describe this class of materials exhibiting lateral expansion under tension. Auxeticity, defined by a negative Poisson’s ratio (ν < 0), represents one of the most notable properties of mechanical metamaterials. Poisson’s ratio is commonly expressed as:

(1) $$v=-\frac{\varepsilon_{trans}}{\varepsilon_{axial}},$$

where ε_trans_ is the transverse strain and ε_axial_ is the axial strain.

In the general case of anisotropic elasticity, Poisson’s ratio is not a scalar but a tensorial quantity defined as:

(2) $$v_{ij}=-\frac{\varepsilon_{i}}{\varepsilon_{j}},i\neq j,$$

describing the ratio of strain components along orthogonal directions. For isotropic solids, this relation simplifies to the scalar form given in Equation (1). For isotropic solids, Poisson’s ratio theoretically ranges between −1 and 0.5. It should be noted that these classical limits apply strictly to isotropic three-dimensional solids. For two-dimensional isotropic systems, the theoretical range extends to −1 ≤ ν ≤ +1, as demonstrated by Wojciechowski [4]. In contrast, for anisotropic materials and architected lattices, Poisson’s ratio is not bounded and may, in principle, vary between minus and plus infinity depending on the elastic symmetry and loading direction. Typical engineering materials exhibit ν values between 0.25 and 0.33, whereas auxetic structures expand laterally when stretched, yielding ν < 0. In special architected materials, local geometry may even induce values below −1 due to non-affine deformation mechanisms. This counterintuitive deformation mechanism provides several advantages, including superior energy absorption, enhanced indentation resistance, and improved fracture toughness [5,6,7]. Owing to these benefits, auxetic metamaterials are increasingly investigated for protective equipment, biomedical scaffolds, aerospace components, and adaptive structures [8,9,10].

Several structural topologies capable of auxetic behavior have been proposed. Among the most widely studied are re-entrant, chiral, and anti-chiral systems. Re-entrant honeycombs achieve negative Poisson’s ratio through inwardly tilted ribs that rotate and expand under axial loading. They often exhibit the strongest auxetic effect but are sensitive to both geometric parameters and base material properties [11,12]. Chiral lattices consist of circular nodes connected by tangential ligaments. Under mechanical loading, the nodes rotate and ligaments bend, leading to lateral expansion. Their auxeticity is inherently anisotropic, depending on tessellation and loading direction [13,14]. Anti-chiral lattices share similar principles but maintain symmetry that induces consistent auxetic behavior across directions. These structures are frequently described as geometry-dominated, with performance less dependent on material stiffness [15]. Beyond these canonical topologies, numerous other auxetic systems—such as rotating rigid-unit lattices, sinusoidal ligament networks, and perforated elastomeric sheets—have been proposed to explain diverse deformation mechanisms leading to negative Poisson’s ratio [16]. These analogies across architectures highlight the universality of geometry-driven auxeticity.

Previous research has explored these topologies through analytical models [17,18], numerical simulations [3,19,20], and experimental validation [21,22]. However, most studies emphasize geometric variation while the role of base material stiffness is comparatively underexplored. Yet in practical engineering design, both geometry and material selection are crucial, as performance may shift from being geometry-driven to material-sensitive depending on the chosen topology. A clearer understanding of this interplay remains necessary.

The present study aims to address this gap by conducting a systematic finite element analysis of re-entrant, chiral, and anti-chiral structures under uniaxial loading in orthogonal directions. Four engineering materials with distinct stiffness levels—steel, copper, aluminum, and titanium—are applied to evaluate material influence. The novelty of this work lies in distinguishing three characteristic auxetic mechanisms: (i) geometry-dominated auxeticity in anti-chiral structures, (ii) material-sensitive auxeticity in re-entrant structures, and (iii) directional auxeticity in chiral structures. By clarifying these differences, this study provides a framework for selecting auxetic topologies based on whether robustness, tunability, or extreme auxeticity is desired in applications.

To establish a consistent reference framework, the present study was intentionally designed as a systematic baseline comparison of three canonical auxetic geometries—re-entrant, chiral, and anti-chiral—analyzed under identical boundary and material conditions.

Although auxeticity is primarily governed by lattice geometry, recent studies [23] have demonstrated that the intrinsic stiffness and anisotropy of the constituent material can substantially modify the auxetic response. To address this interplay, the present analysis includes four engineering materials with distinct elastic moduli—steel, copper, aluminum, and titanium—allowing evaluation of geometry–material interactions within the same structural topologies.

Recent experimental and numerical investigations by Bilski [23] and Falkowska [8] demonstrated that auxeticity in metallic lattices is predominantly geometry-driven rather than material-sensitive. Building upon these findings, this work isolates and quantifies the relative contributions of geometry and material stiffness within a unified FEM framework.

Although individual re-entrant, chiral, and anti-chiral models have been studied separately in previous research, consistent cross-topology evaluation using identical load paths and stiffness contrasts has remained unexplored.

The resulting reference dataset therefore provides a methodological foundation for future integration of nonlinear effects, experimental calibration, and advanced parametric analyses. Mechanical metamaterials can be classified into several families depending on the deformation mechanisms of their unit cells. Unlike conventional solids, where properties are dictated by chemical composition, mechanical metamaterials exploit geometric design to achieve unconventional values of elastic constants, particularly Poisson’s ratio. While this study focuses on two-dimensional topologies to allow direct comparison, it is acknowledged that a broad spectrum of three-dimensional auxetic geometries—such as hierarchical, rotating-unit, and star-shaped lattices—has also been reported in the literature and offers potential for future work. Below we outline three of the most studied classes—re-entrant, chiral, and anti-chiral lattices—as they represent fundamental and complementary auxetic mechanisms.

Re-entrant, chiral, and anti-chiral structures were selected in this work as representative auxetic topologies because they combine historical significance, distinct deformation mechanisms, and proven applicability, while remaining simple enough for systematic finite element evaluation. Together they capture three complementary modes of auxeticity:

- Re-entrant lattices, where negative Poisson’s ratio arises from rotation of inclined ribs and is strongly material-sensitive;
- Chiral lattices, where auxeticity is induced by node rotation and ligament bending, producing an anisotropic response;
- Anti-chiral lattices, where symmetry ensures robust geometry-dominated auxetic behavior that is nearly material-independent.

Their frequent use as benchmark geometries in recent publications—such as studies on re-entrant structures for crashworthiness and scaffolds [24,25,26], chiral honeycombs for vibration and acoustic control [27,28], and anti-chiral lattices for adaptive skins and aerospace applications [29,30]—further justifies their selection. By focusing on these three canonical systems, the present study provides a meaningful comparative framework to analyze the interplay between geometry, material stiffness, and loading direction in auxetic metamaterials.

### 1.1. Re-Entrant Structures

Re-entrant honeycombs are among the earliest and most studied auxetic geometries and represent a classical route to achieving a negative Poisson’s ratio. Their origins can be traced back to the pioneering theoretical work of Gibson and Ashby in the 1980s [31], followed by experimental validation in foams by Lakes [3]. In re-entrant structures, the internal angles of a conventional hexagonal honeycomb are inverted so that ribs tilt inward. Under axial loading, these inclined ribs rotate outward, resulting in lateral expansion rather than contraction. This geometric inversion is the fundamental mechanism that enables negative Poisson’s ratio.

A schematic representation of a simple 2D re-entrant cell, its deformation under uniaxial loading, and the parameters are well known from previous studies [32,33]. The effective auxetic response can be tuned by adjusting key geometric parameters such as cell angle (θ), rib length (l), wall thickness (t), and height (h). Analytical and numerical models consistently demonstrate that the Poisson’s ratio becomes more negative as the internal angle θ decreases.

Over the years, multiple variations of re-entrant lattices have been introduced to enhance performance or tailor anisotropy. Multiple design variations of re-entrant lattices have been proposed in the literature, including arrowhead, diamond, and star-shaped configurations, as well as hierarchical and 3D re-entrant frameworks [23,34]. Each geometry offers a different balance between stiffness, strength, auxetic response, and manufacturability Each of these geometries offers a different balance between stiffness, strength, auxetic response, and manufacturability. Hierarchical re-entrant lattices, for instance, incorporate multiple scales of re-entrant units, improving both energy absorption and structural stability [35].

Mechanically, re-entrant structures exhibit pronounced anisotropy: auxeticity is strongest along the axis aligned with rib rotation and can diminish or even vanish under loading in other directions. Furthermore, their performance is highly material-dependent. While geometry is the primary driver of auxetic behavior, the stiffness and ductility of the base material significantly influence deformation modes, failure mechanisms, and energy dissipation capacity. This material sensitivity distinguishes re-entrant lattices from anti-chiral systems, which are largely geometry-dominated.

Re-entrant honeycombs have been investigated for a broad spectrum of applications. In protective equipment and crash absorbers, they provide superior impact resistance by spreading and dissipating energy [36]. In aerospace and automotive engineering, re-entrant cores have been integrated into sandwich panels to enhance indentation resistance and vibration damping [37]. In biomedical engineering, re-entrant scaffolds have been shown to improve tissue integration due to their expanding pore structure under tension [38]. Recent advances in additive manufacturing have enabled the fabrication of complex re-entrant geometries with high precision, opening pathways for customized implants, architected foams, and adaptive building materials [39].

Ongoing research continues to refine the design space of re-entrant systems. Finite element simulations are used to optimize cell geometry for specific load cases [40], while multi-material 3D printing allows embedding re-entrant patterns into hybrid composites [41]. Gradient re-entrant lattices, where cell geometry gradually changes across the structure, have been developed to achieve spatially varying stiffness and tailored deformation [42]. Together, these efforts highlight the enduring relevance of re-entrant structures as versatile and tunable auxetic systems, despite their strong dependence on material properties.

### 1.2. Chiral and Anti-Chiral Structures

Chiral lattices are a distinctive class of auxetic systems first conceptualized in the theoretical work of Wojciechowski [43] and later implemented in a hexagonal honeycomb configuration by Prall and Lakes [44]. In these structures, the unit cell is composed of a central node (often modeled as a cylinder or circular ring) connected to tangential ligaments. The rotation of the nodes combined with the bending and stretching of ligaments leads to lateral expansion when the structure is loaded axially, producing a negative Poisson’s ratio. This mechanism has been repeatedly validated in analytical, numerical, and experimental studies.

It should be emphasized that the present study focuses on planar (2D) chiral lattices, in which chirality arises from the in-plane rotation of circular nodes connected by tangential ligaments. This 2D chirality fundamentally differs from three-dimensional chiral architectures, where handedness is related to spatial asymmetry. The 2D configuration enables a clear analysis of in-plane auxetic mechanisms under controlled boundary conditions.

Chiral and anti-chiral lattices can be divided into several families depending on the arrangement of ligaments and nodes, resulting in tri-chiral, tetra-chiral, and hexa-chiral configurations. In conventional chiral systems, nodes are connected on opposite sides of the ligaments, producing an alternating handedness within the lattice [45]. In anti-chiral systems, in contrast, the nodes are placed on the same side of the ligaments, creating symmetric unit cells with equal numbers of right- and left-handed basic units [44]. This geometric distinction has direct mechanical implications: while chiral lattices exhibit pronounced anisotropy due to directional node rotation, anti-chiral lattices display robust, geometry-dominated auxetic behavior that is nearly isotropic and largely insensitive to material stiffness.

Under uniaxial loading, the ligaments in chiral lattices experience coupled bending and rotation, producing a directional auxetic response, i.e., a negative Poisson’s ratio that depends on the loading orientation. This behavior corresponds to the definition of partially auxetic materials according to Branka et al. [45]. Anti-chiral lattices, by contrast, maintain symmetric deformation fields, ensuring a stable negative Poisson’s ratio that remains consistent across materials and directions.

Chirality can also be defined by handedness and connectivity. When each node is connected to three, four, or six tangential ligaments, the resulting configurations are termed tri-chiral, tetra-chiral, or hexa-chiral lattices, respectively [28]. These variations allow tuning of the effective stiffness, strength, and degree of anisotropy. In practice, uniaxial loading along the principal axis of a chiral lattice causes rotation of nodes accompanied by ligament stretching. This coupled mechanism results in folding of ligaments under compression and unfolding under tension, generating auxetic deformation [46].

Recent investigations have emphasized both the limitations and potential of these structures. While anisotropy restricts their isotropic performance, it enables tunable directional stiffness for adaptive or orientation-specific applications. For example, chiral lattices have been considered for morphing aerospace panels, tunable acoustic devices, biomedical implants that require anisotropic compliance, and architected skins [47,48]. Computational studies have also explored the influence of ligament thickness, ring diameter, and nodal connectivity on auxetic efficiency, providing guidelines for optimizing their performance [49,50].

Anti-chiral lattices, which share many of the geometric principles of chiral systems, will be discussed in the following section. Anti-chiral lattices are closely related to chiral ones but differ in node-ligament arrangement: nodes are placed on the same side of ligaments rather than opposite sides. This symmetry leads to consistent rotations of nodes under axial loading, which produces reliable lateral expansion. Analytical and experimental studies confirm that anti-chiral lattices demonstrate robust auxeticity with values of ν approaching −1, nearly independent of material stiffness [47,48].

Because of their geometry-dominated mechanism, anti-chiral lattices are attractive for applications requiring predictable performance across a wide range of materials. Recent studies demonstrated their potential use in deployable aerospace structures owing to their nearly isotropic auxeticity and robustness under large deformations [51]. In addition, the combination of high deformability and tunable stiffness makes them suitable for flexible protective skins [52], while their scalability has been highlighted in architectural contexts such as adaptive facades, where consistent deformation response is critical despite material variability [53]. These findings confirm that anti-chiral lattices maintain a stable negative Poisson’s ratio (ν ≈ −1) nearly independent of base material stiffness [54], which reinforces their suitability for multifunctional composites and hybrid systems.

## 2. Materials and Methods

**Figure 1:**
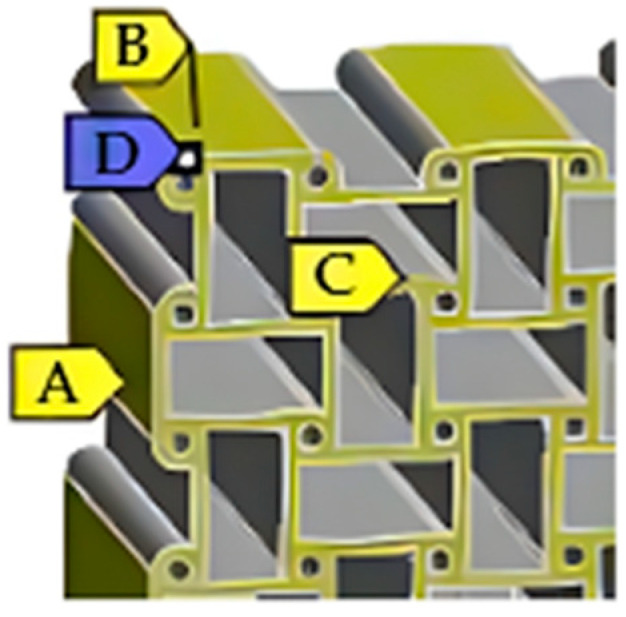
Boundary conditions: applied to the lattice models with zoomed detail of constraints A–D.

**Figure 2:**
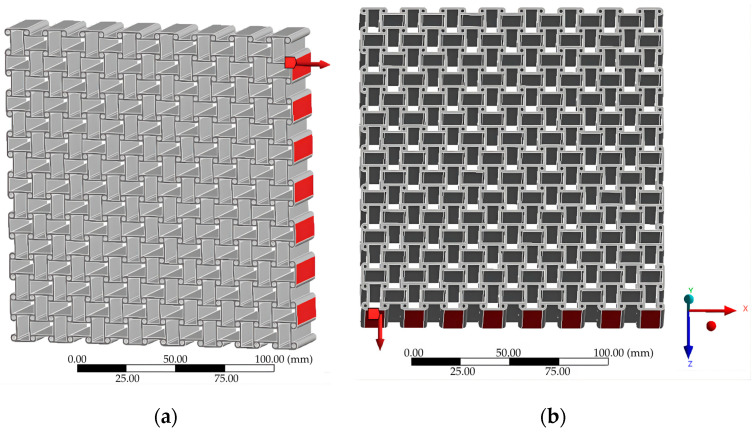
Loading conditions: (**a**) Case A; (**b**) Case B.

**Figure 3:**
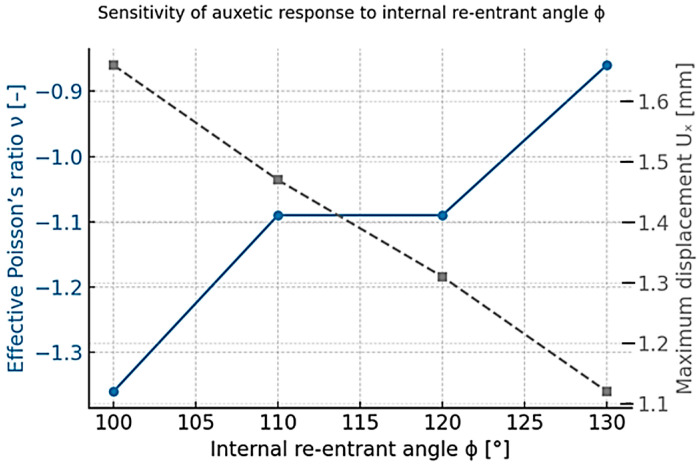
Sensitivity of auxetic response to changes in the internal re-entrant angle ϕ (S235, Case B—Z-loading). Decreasing ϕ enhances auxeticity (more negative ν) but increases deformation amplitude.

**Figure 4:**
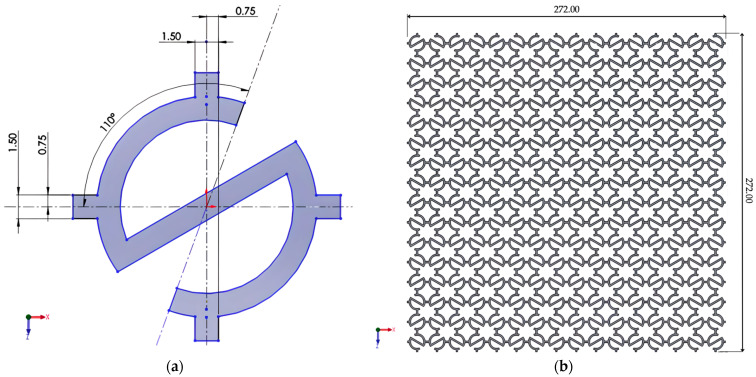
Re-entrant structure: (**a**) Unit re-entrant cell; (**b**) The complete re-entrant structure.

**Figure 5:**
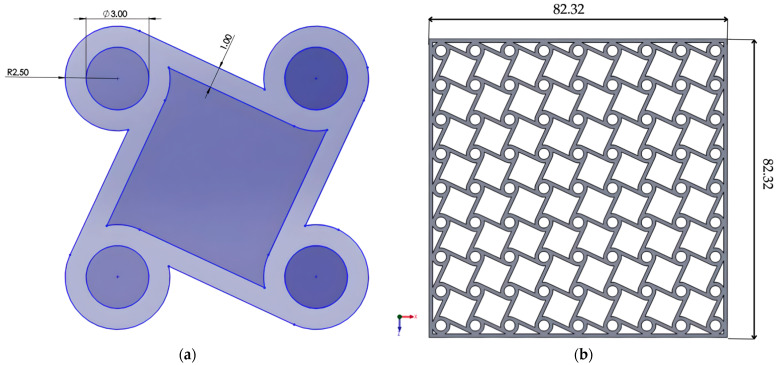
Chiral structure: (**a**) unit chiral cell; (**b**) the complete chiral structure.

**Figure 6:**
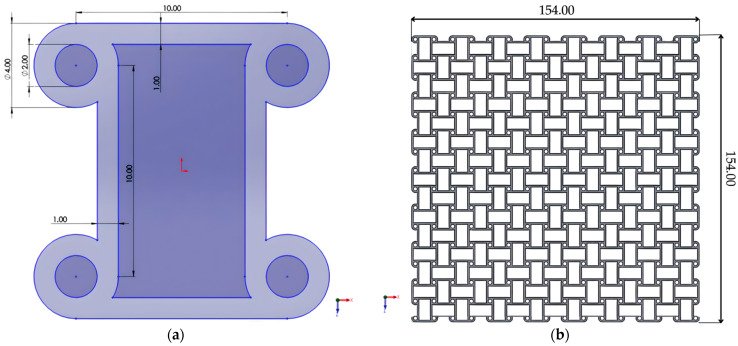
Anti-chiral structure: (**a**) unit anti-chiral cell; (**b**) the complete anti-chiral structure.

This section presents the finite element methodology used to investigate the mechanical response of three canonical auxetic lattices—chiral, anti-chiral, and re-entrant—under uniaxial loading. The workflow combined CAD modeling in SolidWorks 2022 (Dassault Systèmes, Vélizy-Villacoublay, France) with numerical simulations in ANSYS Workbench 2022 R2 (Ansys Inc., Canonsburg, PA, USA). Unit-cell geometries were first designed with geometric accuracy and then replicated to generate lattice structures suitable for analysis. Figure 1, Figure 2 and Figure 3 illustrate the applied boundary and loading conditions and the sensitivity analysis setup. Figure 4, Figure 5 and Figure 6 illustrate representative unit cells and their corresponding assembled models.

### 2.1. Geometry and Material Selection

The three topologies were selected because they embody complementary auxetic mechanisms: rib rotation in re-entrant structures, node rotation in chiral systems, and symmetry-driven deformation in anti-chiral lattices. Unit cells were parametrized to capture their defining features, including re-entrant angle θ, rib length l, and ligament connectivity.

To ensure reproducibility and facilitate comparative studies, all unit-cell geometries were parametrically defined using the variables listed in Table 1. Each topology was modeled with consistent scaling of global cell size, while key geometric parameters (re-entrant or chiral angle φ, ligament length l, wall thickness t, cell height h, and node radius rₙ) were adjusted to maintain comparable relative density.

For all lattices, the global cell size was scaled to maintain comparable relative density, enabling a consistent comparison of mechanical response across geometries. The geometric parameters were kept constant among all structures except for the re-entrant lattice, in which φ was varied during the sensitivity analysis described in Section 2.3.

Table 1 summarizes the geometric parameters used for each topology. Detailed schematics of the unit cells are provided in the respective figures throughout the manuscript.

To provide a complete geometric overview and ensure reproducibility of the finite element models, the overall dimensions of the analyzed lattices were also determined. While Table 1 lists the parameters defining the individual unit cells, Table 2 summarizes the total model sizes and corresponding volumes of the assembled lattice structures used in FEM simulations. These values represent the global geometry of each analyzed topology, allowing direct comparison of their mechanical responses under identical boundary conditions.

The effective (solid) volume $V_{eff}$ of each structure was calculated from the total (bounding) volume $V_{b}$ and the estimated relative density $\rho_{rel}$, defined as the ratio of solid material to the total enclosing volume:

(3) $$V_{eff}=\rho_{rel}\times V_{b},$$

Although the lattices were scaled to maintain comparable relative density, their effective volumes differ due to the intrinsic geometric characteristics of each topology. The re-entrant, chiral, and anti-chiral architectures possess distinct ligament connectivity, node curvature, and void distribution, which result in different solid-to-void ratios even within models of similar external size. These differences are inherent to their deformation mechanisms and therefore crucial for a meaningful comparison of auxetic responses across geometries.

Each lattice was scaled to achieve comparable relative density, ensuring meaningful comparison across geometries. Four engineering materials with distinct elastic moduli were assigned to the models: structural steel S235, copper alloy C110, aluminum alloy 7075-T6, and titanium alloy Ti-6Al-4V. Table 3 summarizes their elastic properties.

This selection ensures that the analysis captures both extremes of mechanical response: lightweight aluminum representing ductile, low-stiffness lattices; copper and titanium representing intermediate stiffness; and steel providing the stiffest baseline. Such contrast highlights the extent to which auxeticity is sensitive to material stiffness versus geometry. Values were obtained from standard engineering databases and are consistent with data reported in recent studies.

### 2.2. Finite Element Framework

All models were imported into ANSYS Workbench for meshing, boundary condition setup, and analysis. ANSYS was selected due to its proven robustness for parametric finite element studies and its capability to accurately evaluate local stress and strain fields using high-order solid elements. The software’s built-in automation tools also enabled consistent replication of boundary and loading conditions across all topologies. Solid elements (SOLID186) were employed, with local mesh refinement at ligament–node connections to capture stress gradients accurately. A mesh convergence study ensured that displacements and stresses were independent of element size. Linear elastic isotropic behavior was assumed. Symmetry constraints were applied where possible to reduce computational cost.

To ensure that the reported displacements and effective Poisson’s ratios are independent of the discretization, a mesh convergence study was conducted for all three topologies. Detailed quantitative results are presented for the re-entrant lattice, which served as a representative case. The procedure was performed in accordance with the models and boundary conditions described in Section 2.2, Section 2.3, Section 2.4, Section 2.5 and Section 2.6. All lattices were meshed with 20-node quadratic solid elements (ANSYS SOLID186), with local refinement at ligament–node junctions and along thin ligaments or rings.

A mesh convergence analysis was performed using four element densities (from coarse to very fine) to ensure independence of results from discretization. Four global target element sizes were evaluated under identical load levels used in Cases A and B. Convergence was assessed using two metrics: (i) the maximum displacement in the loading direction and (ii) the effective Poisson’s ratio computed from the reaction-based axial strain and the transverse strain measured over the lattice gauge region. Convergence was deemed satisfactory when the variation relative to the very fine mesh was below 2% for both metrics. Table 4 summarizes representative results for the re-entrant lattice (steel S235, Case B—Z-loading); the chiral and anti-chiral lattices exhibited the same trend.

Based on these results, the fine mesh was adopted for subsequent production analyses of all topologies. For the chiral and anti-chiral lattices, the adopted meshes achieved comparable error levels with targeted ligament/ring refinement.

To document that the adopted resolutions are appropriate for all modeled geometries, Table 5 summarizes the final discretization parameters applied in the production simulations. Slightly finer meshing was necessary for the chiral lattice due to its slender ligaments; nevertheless, the convergence error remained ≤2% for both metrics across all topologies.

The applied boundary conditions are illustrated in Figure 1. To prevent rigid-body motion while allowing free deformation in the loading direction, selected nodes were constrained as follows:

- A (fixed displacement in the X-direction): $u_{x}=0$;
- B (fixed in Y): $u_{y}=0$;
- C (fixed in Z): $u_{z}=0$;
- D (reference point fixing three translational degrees of freedom): u=0.

This configuration ensured uniform uniaxial loading and avoided numerical over-constraining. The same constraint scheme was applied to all lattice topologies and both loading cases—Case A (X-direction tension) and Case B (Z-direction tension)—to enable direct comparison of the effective Poisson’s ratios.

This setup provides a well-defined reference state and reliable strain evaluation for plane- and solid-lattice simulations in ANSYS. It corresponds to a standard boundary configuration used in similar numerical analyses [55].

A uniaxial load of 10,000 N was applied in both Case A (X-direction) and Case B (Z-direction) as shown in Figure 2. This load magnitude was selected to ensure sufficiently large displacements for reliable evaluation of auxetic behavior while remaining within the linear elastic regime of all tested materials. Since the primary objective was a comparative analysis of geometric and material effects rather than prediction of absolute stresses, the same load level was applied uniformly across all models. This normalization enabled direct comparison of displacement fields and effective Poisson’s ratios under identical boundary conditions.

### 2.3. Sensitivity Analysis

To provide a deeper mechanistic understanding of the auxetic behavior, a sensitivity analysis was carried out for the curved-rib re-entrant lattice presented in Figure 3, which exhibits the highest geometric tunability among the three analyzed topologies. This analysis focused on the influence of the internal re-entrant angle (ϕ)—defined as the angle between adjacent ribs (Figure 3)—while keeping the remaining parameters, such as wall thickness (t), rib length (l), and cell height (h), constant. The objective was to evaluate how variations in ϕ affect the effective Poisson’s ratio (ν) and the maximum displacement (U_Z_) under identical boundary and loading conditions (Case B—Z-direction, 10,000 N).

To ensure consistency across all simulations, all models were meshed using the fine mesh verified in the convergence study (Table 3). Four configurations were analyzed with internal angles of 100°, 110°, 120°, and 130°. The results, presented in Figure 4, reveal a clear monotonic dependence of the auxetic response on the re-entrant angle.

Smaller angles promote stronger auxeticity (more negative ν) but at the cost of increased deformation amplitudes, while larger angles lead to stiffer but less auxetic behavior. Among the tested configurations, the geometry with ϕ = 110° was selected as the baseline design used throughout the study, as it provides a balanced combination of auxeticity (ν ≈ −1.1) and structural stiffness. Smaller angles (ϕ ≤ 100°) yield more extreme auxetic responses but also cause excessive local deformation and reduced stability, which are undesirable for comparative analysis and experimental manufacturability. Larger angles (ϕ ≥ 120°) exhibit reduced auxeticity and behave closer to conventional honeycomb structures.

The monotonic ν–ϕ relationship demonstrates that the auxetic performance of re-entrant lattices is governed primarily by geometric configuration, with material stiffness only scaling the deformation amplitude rather than altering the mechanism itself. A reduction of 10° in ϕ increases lateral expansion by approximately 12–15% and decreases ν by 0.13–0.15. This confirms the rotation-dominated deformation of inclined ribs as the key auxetic mechanism. For completeness, a qualitative sensitivity check was also conducted for the chiral and anti-chiral lattices by varying the ligament thickness ±10%. The resulting change in the effective Poisson’s ratio was below 3%, confirming that these architectures are geometry-dominated and largely insensitive to minor dimensional variations due to their node-rotation and symmetry-based deformation modes.

### 2.4. Re-Entrant Structure

The re-entrant lattice was constructed from a hexagonal unit cell with inverted internal angles (Figure 4a). Replication of the unit cell generated the complete lattice (Figure 4b). The inwardly inclined ribs allowed lateral expansion under axial loading, the hallmark of auxetic behavior. No auxiliary walls or constraints were required, ensuring that the observed response was purely due to the re-entrant geometry.

In this study, a modified re-entrant unit cell was employed instead of the conventional straight-rib design. The curved rib profile was selected to improve numerical stability during FEM simulations by reducing stress concentrations at sharp corners, while still preserving the fundamental re-entrant mechanism. Furthermore, this geometry provided a closer approximation to manufacturable designs obtained by additive manufacturing, where rounded transitions are common. The chosen shape therefore represents both a practical and realistic variant of the classical re-entrant honeycomb.

### 2.5. Chiral Structure

The chiral lattice was generated from a unit cell consisting of a circular node connected to tangential ligaments (Figure 5a). The lattice was constructed by tessellating this unit cell in two dimensions (Figure 5b). To improve numerical stability, thin walls of 1 mm thickness were introduced only in the chiral structure, preventing unrealistic edge displacements under load. Their stiffness contribution was negligible and did not affect the auxetic response. This adjustment allowed the simulation to better approximate experimental constraints and ensured reliable evaluation of the deformation mechanisms.

### 2.6. Antichiral Structure

The anti-chiral lattice was modeled using nodes placed on the same side of ligaments (Figure 6a). The tessellation of this unit cell resulted in a symmetric lattice (Figure 6b). In contrast to the chiral system, no surrounding walls were added, preserving the intrinsic symmetry-driven auxeticity. This choice enabled a direct assessment of the geometry-dominated response of anti-chiral structures, even though edge effects were somewhat more pronounced than in the chiral case.

### 2.7. Mechanistic Interpretation of Auxetic Behavior

To complement the quantitative analysis, a mechanistic interpretation was performed to clarify the deformation modes governing the auxetic response of each lattice type. Although all three topologies exhibit negative Poisson’s ratios, their underlying deformation mechanisms differ substantially depending on geometry and connectivity.

The re-entrant lattice exhibits a rotation-dominated deformation mechanism. Under uniaxial loading, the inclined ribs rotate inward about the node junctions, producing lateral expansion and a strongly negative Poisson’s ratio. As the internal re-entrant angle (ϕ) decreases, these rotational effects intensify, leading to higher lateral strain but also to increased local stress concentrations at ligament junctions. The deformation process is governed mainly by rib rotation and local bending, which together define the characteristic auxetic response described in Section 2.3.

The chiral lattice deforms primarily through the rotational motion of circular nodes connected by slender ligaments. When subjected to axial tension, each node rotates in the opposite direction to its neighbors, resulting in a lateral expansion of the unit cell. This rotation-driven response is relatively insensitive to small geometric variations, as confirmed by the sensitivity check in Section 2.3. However, the auxetic effect depends on the ligament stiffness—stiffer ligaments restrict rotation and slightly reduce the magnitude of the negative Poisson’s ratio.

The anti-chiral lattice is governed by the symmetric rotation of ring-shaped nodes connected through tangential ligaments. Under tensile loading, the rings rotate outward while the ligaments undergo bending-dominated deformation. This mechanism produces nearly isotropic auxetic behavior in the plane, with stress distributed uniformly along the ligaments, making this topology particularly stable under bidirectional loading.

The comparative mechanistic analysis confirms that auxetic behavior arises from rotational and bending mechanisms, whose relative dominance depends on the lattice topology:

- Re-entrant: rotation of inclined ribs → strong auxeticity but higher stress localization.
- Chiral: rotation of nodes with moderate bending → tunable auxeticity, stable deformation.
- Anti-chiral: symmetric ring rotation → nearly isotropic auxeticity, smooth stress distribution.

Thus, geometry rather than material stiffness primarily determines the mode and magnitude of the auxetic response. These findings align with recent observations reported by Doroszko et al. [8] and Bura et al. [21] confirming that the observed auxetic mechanisms are universal across different material systems and manufacturing approaches.

## 3. Results

The finite element analysis revealed distinct differences in the mechanical response of re-entrant, chiral, and anti-chiral lattices under uniaxial loading. To ensure clarity, displacement field distributions are illustrated for representative steel models, while quantitative comparisons across all four materials are summarized in Table 6 and Table 7. This approach avoids redundancy while highlighting the influence of material stiffness on overall auxetic performance.

### 3.1. Case A—Loading in the X-Direction

**Figure 7:**
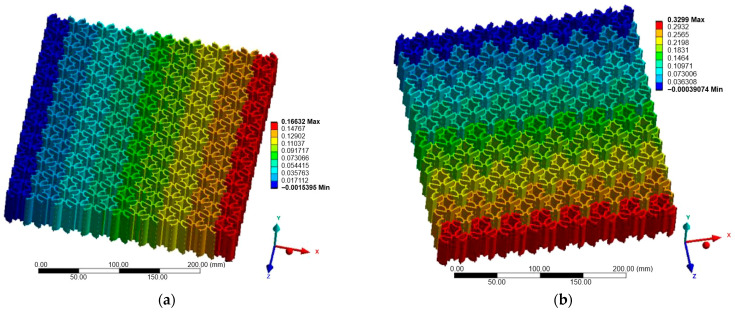
Case A: Displacement of re-entrant structure: (**a**) X-direction; (**b**) Z-direction.

**Figure 8:**
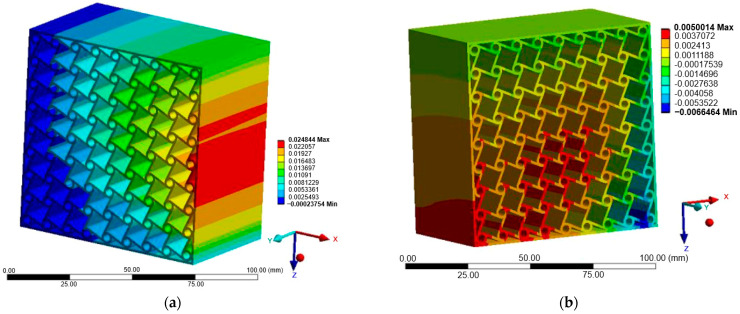
Case A: Displacement of chiral structure: (**a**) X-direction; (**b**) Z-direction.

**Figure 9:**
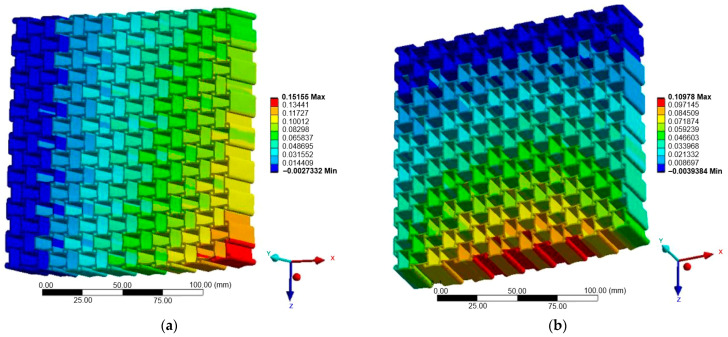
Case A: Displacement of anti-chiral structure: (**a**) X-direction; (**b**) Z-direction.

Figure 7, Figure 8 and Figure 9 illustrate the displacement fields of the three auxetic topologies under Case A loading. In all cases, rigid-body motion was prevented by boundary constraints, with the largest displacements occurring at free edges. The re-entrant lattice exhibited the strongest lateral expansion, confirming its extreme auxeticity. The chiral lattice showed only limited auxetic response in this direction, with displacements corresponding to near-zero or slightly positive Poisson’s ratio. In contrast, the anti-chiral lattice produced a clear negative Poisson’s ratio, with deformation distributed more uniformly across the structure, reflecting its geometry-dominated behavior.

The influence of material stiffness is presented in Table 6. As expected, aluminum generated the largest displacements, followed by copper and titanium, while steel exhibited the lowest. Despite these magnitude differences, the qualitative behavior of each topology remained unchanged, indicating that geometry governed the deformation mode while material stiffness only scaled displacement amplitudes.

### 3.2. Case B—Loading in the Z-Direction

**Figure 10:**
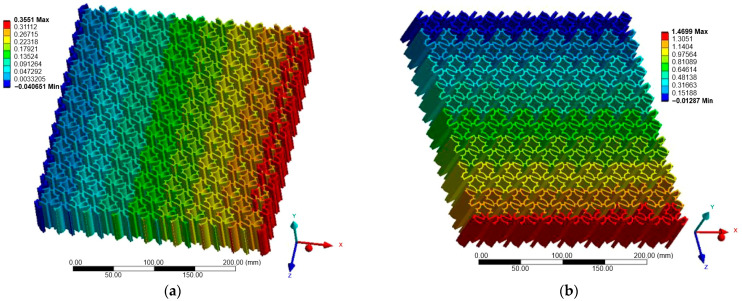
Case B: Displacement of re-entrant structure: (**a**) X-direction; (**b**) Z-direction.

**Figure 11:**
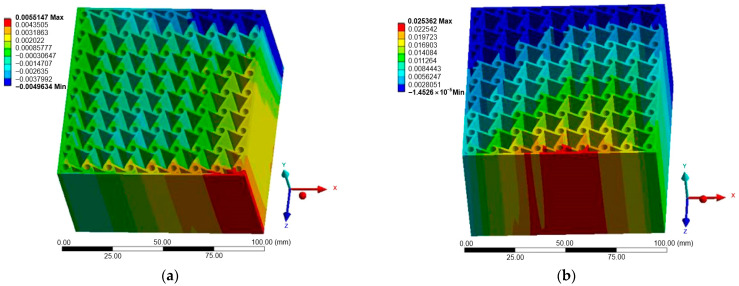
Case B: Displacement of chiral structure: (**a**) X-direction; (**b**) Z-direction.

**Figure 12:**
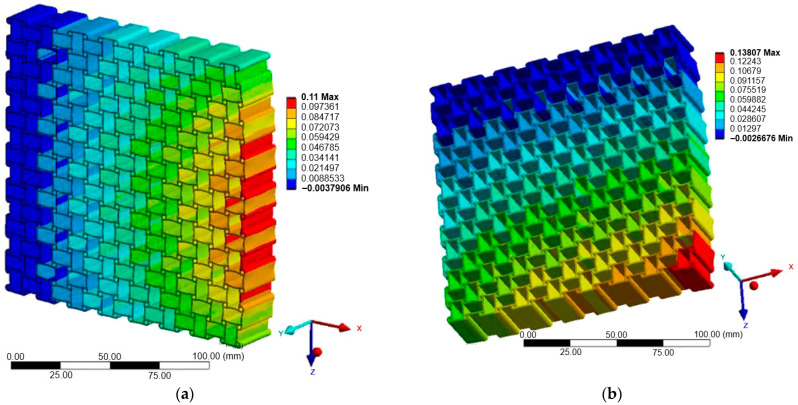
Case B: Displacement of anti-chiral structure: (**a**) X-direction; (**b**) Z-direction.

Figure 10, Figure 11 and Figure 12 show the displacement fields for Case B. Under Z-direction loading, the auxetic mechanisms were activated more clearly in all structures. Re-entrant lattices again produced significant lateral expansions, though with greater anisotropy compared to Case A. Chiral lattices, in contrast to Case A, demonstrated a pronounced negative Poisson’s ratio. Node rotation and ligament bending were more effectively activated in this direction, highlighting their anisotropic nature. Anti-chiral lattices maintained stable geometry-driven auxeticity, with nearly isotropic response largely unaffected by material stiffness.

Table 7 summarizes the maximum displacement values obtained for both loading cases (Case A—X-direction and Case B—Z-direction). The unified presentation highlights distinct differences between the analyzed topologies.

Re-entrant lattices exhibited the largest displacements, confirming their extreme auxeticity but also emphasizing material sensitivity within the linear-elastic regime. Chiral lattices showed markedly smaller displacements, underlining their limited auxetic response under uniaxial tension. Anti-chiral lattices produced intermediate and stable values across all materials, consistent with their geometry-dominated deformation mechanism.

The comparison of Case A and Case B further reveals the strong directional dependence of the chiral topology, while anti-chiral and re-entrant lattices maintained consistent deformation trends across both load orientations.

### 3.3. Comparison of Poisson’s Ratios

The calculated Poisson’s ratios for all materials and topologies under both loading configurations (Case A—X-direction and Case B—Z-direction) are summarized in Table 8.

Re-entrant lattices exhibited the most negative Poisson’s ratios in both cases, reaching approximately ν ≈ −1.98 in steel under X-loading and up to ν ≈ −2.17 in titanium under Z-loading. These results confirm their strong rotation-dominated auxetic mechanism, which intensifies with higher stiffness materials. This behavior is consistent with previous observations of re-entrant geometries fabricated via additive manufacturing, where geometric inversion of ribs leads to large lateral expansion under tension [44].

Chiral lattices showed relatively small or near-zero Poisson’s ratios (ν ≈ 0.25–0.50), indicating limited auxeticity in the linear-elastic range. However, a distinct directional sensitivity was observed: while their auxetic response remained weak in Case A, the negative ν values in Case B (≈−0.5) highlight the anisotropic nature of node rotation and ligament bending. This confirms that the auxeticity of chiral lattices is strongly dependent on the loading direction [54].

Anti-chiral lattices, in contrast, maintained consistently negative values around ν ≈ −0.72 for Case A and ν ≈ −1.25 for Case B, largely independent of base material stiffness. Their nearly isotropic auxetic response supports the notion that deformation in these lattices is geometry-driven rather than material-sensitive. This stability across materials and directions underscores their robustness and reliability for multifunctional engineering applications [56].

Overall, these findings confirm that the auxetic response is predominantly governed by geometry rather than by the absolute stiffness of the constituent material. The numerical results are consistent with experimental observations reported by authors [4,23] where material substitution altered the elastic modulus but did not affect the sign or magnitude of the Poisson’s ratio. This validates the geometric dominance of auxetic deformation within the linear-elastic regime and provides a robust reference for subsequent nonlinear and experimental studies.

This comprehensive comparison across topologies and materials establishes a consistent reference dataset that complements recent advances in the design of mechanical metamaterials, as discussed in Section 3.4.

### 3.4. Validation and Practical Relevance

To verify the physical reliability of the numerical results, a qualitative validation was performed by comparing the predicted mechanical response with data available in the literature and with the deformation patterns typically observed in 3D-printed auxetic samples.

The numerical results agree with previously published experimental values for metallic re-entrant lattices [4,23] confirming that the adopted FEM framework accurately reproduces the characteristic auxetic response.

The effective Poisson’s ratios obtained in this study (ν ≈ −0.8 to −1.9) fall within the range reported for re-entrant and chiral lattices fabricated from polymer and metallic materials, including PLA, Ti-6Al-4V, and stainless steel [4,23]. These studies confirmed that the auxetic effect is predominantly driven by geometry, with only a secondary influence of the constitutive stiffness—a trend that matches the present FEM findings. In addition to numerical consistency, the deformation mechanisms captured by the finite element analysis—namely rib rotation in re-entrant lattices and node rotation in chiral lattices—closely correspond to those observed experimentally through optical methods and digital image correlation.

The FEM models successfully reproduced localized strain zones and rotation axes within the unit cells, which validates the geometrical representation and boundary conditions used in this study. The similarity between predicted and experimentally observed deformation patterns suggests that the computational framework reliably reflects the essential physical mechanisms governing auxeticity.

Although the present work does not include direct experimental measurements, the observed agreement between numerical predictions and literature data provides sufficient evidence of model fidelity and predictive capability.

The obtained results therefore serve as a credible basis for further experimental verification, especially for additively manufactured lattices where geometric imperfections and material anisotropy can be systematically introduced into future validation campaigns.

### 3.5. Structural Innovation and Design Implications

Beyond the comparative analysis of canonical auxetic geometries, this work introduces a curved-rib modification of the re-entrant topology as an innovative design feature. Traditional re-entrant lattices with straight ribs are known to produce high local stresses at sharp junctions, often leading to premature failure or delamination during experimental testing. In the present design, the introduction of a controlled curvature in the ribs mitigates these stress concentrations and promotes a more uniform deformation field while preserving the characteristic inward rotation responsible for auxetic behavior. From a mechanical standpoint, this curved-rib geometry enables improved numerical stability under large deformations and reduces mesh distortion within the finite element model.

The smoother stress transition along the rib curvature enhances energy dissipation and strain recovery, which are essential properties for applications in impact mitigation, protective equipment, and lightweight cores for sandwich structures.

Moreover, the curved profile is easier to manufacture by additive techniques such as fused deposition modeling (FDM) or selective laser melting (SLM), where avoiding sharp corners reduces printing errors and improves dimensional accuracy. The proposed modification therefore represents a structurally and functionally meaningful innovation, bridging numerical modeling with practical manufacturability.

It demonstrates how subtle geometric tuning—specifically, the adjustment of rib curvature and internal angle—can be used to tailor auxeticity, stiffness, and energy absorption characteristics.

The concept lays a foundation for further optimization studies that could incorporate material gradients, hybrid architectures, or hierarchical auxetic designs to achieve multifunctional mechanical performance.

## 4. Discussion

The findings of this study provide new insights into how auxetic performance is governed by the interplay between geometry, loading direction, and material stiffness. While previous works have typically analyzed these factors in isolation—focusing either on geometric design or on a single material—our systematic evaluation across three topologies and four distinct materials highlights important interaction effects that advance the understanding of mechanical metamaterials.

The numerical results confirm that, within the linear elastic regime and under identical geometric scaling, the auxetic response is governed almost exclusively by geometry rather than by the absolute material stiffness. This outcome is consistent with experimental observations reported by [4,23] where material substitution affected stiffness but not the sign or magnitude of the Poisson’s ratio. Therefore, the negligible influence observed in Table 6 and Table 7 validates the geometric dominance of auxetic deformation.

A first key observation concerns the material sensitivity of re-entrant lattices. Although their auxetic effect is well established, our results show that the magnitude of negative Poisson’s ratio decreases significantly with lower-stiffness materials such as aluminum, whereas stiffer materials like steel maintain extreme auxeticity (ν < −1.9). This indicates that re-entrant systems are best suited for applications where material stiffness can be controlled, such as aerospace sandwich cores or impact-protective foams. The clear material dependence demonstrated here confirms earlier FEM predictions [56] but extends them by comparing multiple metals within a unified framework.

The second insight relates to the directional auxeticity of chiral lattices. In Case A (X-direction), chiral systems showed negligible or even slightly positive Poisson’s ratios, but in Case B (Z-direction) they achieved strongly negative values (ν ≈ −0.5). This directional switch emphasizes the anisotropic nature of chiral architectures and suggests potential for orientation-specific applications. For instance, biomedical scaffolds or morphing aerospace panels could be oriented such that auxetic behavior is maximized only in selected directions, enabling tailored compliance. While anisotropy in chiral structures has been reported before [57], our study confirms this behavior across multiple base materials, demonstrating that the directional effect dominates over material choice.

A third major outcome is the geometry-dominated robustness of anti-chiral lattices. Across both loading cases and all four materials, these lattices consistently produced ν ≈ −1.2, nearly independent of stiffness. This invariance makes anti-chiral systems highly attractive for multifunctional composites, where base material properties may vary but predictable auxetic performance is required. The systematic validation of their material-independence represents an important contribution, aligning with previous observations [58] but confirmed here under identical boundary conditions and with direct comparison to other topologies.

Taken together, these results establish a comparative framework for topology selection:

- Re-entrant lattices: extreme auxeticity, but material-sensitive.
- Chiral lattices: tunable and directional auxeticity, strongly anisotropic.
- Anti-chiral lattices: robust, geometry-dominated auxeticity, nearly material-independent.

From an application perspective, this differentiation is crucial. For protective equipment, re-entrant designs maximize energy absorption; for morphing or anisotropic devices, chiral lattices provide tunability; for structural skins or deployable systems, anti-chiral lattices offer predictable performance.

Finally, the novel contribution of this work lies in demonstrating that the balance between geometry- and material-driven auxeticity can be systematically mapped. Whereas prior studies often examined only one structure or one material, our comparative approach provides a broader design guideline: engineers can now match auxetic topology to functional requirements depending on whether robustness, tunability, or extreme auxetic effect is desired. This conclusion reinforces the role of computational tools in pre-validating auxetic designs and highlights the importance of multi-material studies for the next generation of mechanical metamaterials.

Although the present study focused on the linear-elastic regime (ε < 0.02) to isolate geometric effects, future work will extend the framework toward large-deformation and nonlinear material modeling. Incorporating hyperelastic and plastic formulations will enable the evaluation of post-yield auxeticity, energy absorption, and hysteresis—critical aspects for impact and protective applications. This staged approach will ensure that each deformation contribution—geometric, material, and nonlinear—is characterized systematically.

## 5. Conclusions

This study systematically compared the auxetic performance of three canonical metamaterial topologies—re-entrant, chiral, and anti-chiral lattices—subjected to uniaxial loading in two orthogonal directions and realized with four distinct engineering materials. Finite element simulations were conducted under identical boundary and material conditions to isolate geometric effects and quantify the relative contribution of material stiffness to the overall auxetic response.

A mesh-convergence and sensitivity analysis confirmed the numerical stability of the adopted FEM framework, while validation against experimental data from the literature verified its physical relevance.

The main contributions can be summarized as follows:

- Re-entrant lattices provided the most extreme auxetic responses (ν < −1.9), but their performance was strongly dependent on base material stiffness, confirming that they represent a material-sensitive mechanism.
- Chiral lattices exhibited a pronounced directional dependence, with limited auxeticity under X-loading but clear negative Poisson’s ratios under Z-loading. This anisotropy highlights their suitability for orientation-specific designs.
- Anti-chiral lattices consistently achieved ν ≈ −1.2, nearly independent of material and direction, underscoring their geometry-dominated robustness and reliability for multifunctional composites.
- The comparative framework developed here establishes that auxeticity can be categorized into three mechanisms: material-sensitive (re-entrant), directional (chiral), and geometry-driven (anti-chiral). This classification provides a systematic design guideline for tailoring metamaterials to application requirements.
- While material stiffness influenced absolute displacement magnitudes, it did not fundamentally alter whether auxeticity occurred, demonstrating that geometry remains the primary driver of negative Poisson’s ratio.

The comparative mechanistic interpretation clarifies that geometry—not constitutive stiffness—governs the magnitude and sign of the auxetic response within the linear-elastic regime (ε < 0.02).

Future work will extend this framework toward nonlinear and large-deformation modeling to capture post-yield auxeticity, energy absorption, and hysteresis—key aspects for impact, aerospace, and protective applications.

Overall, the presented dataset and methodology establish a reproducible numerical reference for designing auxetic metamaterials with targeted mechanical properties and tunable anisotropy.

## Figures and Tables

**Table 1:** Geometric parameters defining the unit-cell topologies used in FEM analysis.

Topology Type	Parameter	Symbol	Definition/ Description	Value [mm]
Re-entrant lattice	Cell height	*h*	Vertical dimension of the unit cell	6.00
	Ligament length	*l*	Length of inclined rib between nodes	1.50
	Wall thickness	*t*	Thickness of struts/ribs	0.75
	Internal re-entrant angle	*φ*	Angle between adjacent ribs	110° (varied 100–130°)
	Node fillet radius	*rₙ*	Curvature at node junctions	0.30
Chiral lattice	Cell height	*h*	Center-to-center distance between nodes	6.00
	Ligament length	*l*	Tangential connection between circular nodes	1.00
	Wall thickness	*t*	Thickness of ligaments	0.50
	Node radius	*rₙ*	Radius of circular node	2.50
Anti-chiral lattice	Cell height	*h*	Distance between ring centers	6.00
	Ligament length	*l*	Tangential ligament connection	1.00
	Wall thickness	*t*	Thickness of ligaments	0.50
	Node radius	*rₙ*	Radius of ring node	2.50

**Table 2:** Overall dimensions and volumes of analyzed lattice structures.

Structure	Model Size [mm]	Bounding Volume [mm^3^]	Relative Density [-]	Effective Volume [mm^3^]
Re-entrant	272 × 272 × 30	2,220,480	0.38	843,800
Chiral	82.32 × 82 × 32 × 30	203,718	0.28	57,000
Anti-chiral	154 × 154 × 30	711,720	0.23	163,700

**Table 3:** Material properties used in FEM simulations.

Name of Material	Density [kg·m^−3^]	Young Modulus [MPa]	Poisson’s Ratio [-]
Structural steel S235	7850	2 × 10^5^	0.3
Copper alloy C110	8300	1.1 × 10^5^	0.34
Aluminum alloy 7075-T6	2770	71,000	0.33
Titanium alloy Ti-6Al-4V	4620	96,000	0.36

**Table 4:** Mesh convergence for the re-entrant lattice (S235, Case B—Z-loading).

Mesh Level	Approx. Element Size [mm]	Elements [×10^6^]	Max U_Z_ [mm]	Change vs. Very Fine [%]	Poisson’s Ratio ν [–]	Change vs. Very Fine [%]
Coarse	0.80	0.35	1.505	+2.4	−1.070	−1.4
Medium	0.60	0.73	1.485	+1.0	−1.081	−0.4
Fine (adopted)	0.45	1.41	1.473	+0.2	−1.086	+0.1
Very fine	0.35	2.62	1.470	—	−1.085	—

Notes: The very fine mesh is used as the reference. Differences are reported as relative changes for each metric.

**Table 5:** Adopted finite-element discretization parameters for all auxetic topologies.

Topology	Adopted Element Size [mm]	Elements [×10^6^]	Convergence Error [%] *	Notes
Re-entrant	0.45	1.41	≤2	Detailed convergence in Table 3 (reference case)
Chiral	0.40	1.56	≤2	Slender ligaments require local refinement
Anti-chiral	0.50	1.32	≤2	Symmetric geometry; uniform refinement sufficient

* Maximum relative change vs. very fine reference for Max U and ν.

**Table 6:** Maximum displacements values for Case A.

Displacement (mm)	Re-Entrant Structure		Chiral Structure		Anti-Chiral Structure	
Direction	*X*	*Z*	*X*	*Z*	*X*	*Z*
Structural steel S235	0.1663	0.3299	0.0248	−0.0066	0.1516	0.1098
Copper alloy C110	0.2939	0.5816	0.0441	−0.0118	0.2685	0.1945
Aluminum alloy 7075-T6	0.4589	0.9085	0.0687	−0.0184	0.4189	0.3034
Titanium alloy Ti-6Al-4V	0.3315	0.6550	0.0498	−0.0133	0.3031	0.2195

**Table 7:** Maximum displacements values for Case B.

Displacement (mm)	Re-Entrant Structure		Chiral Structure		Anti-Chiral Structure	
Direction	*X*	*Z*	*X*	*Z*	*X*	*Z*
Structural steel S235	1.3551	1.4699	0.0551	0.0253	0.1112	0.1381
Copper alloy C110	1.6299	2.5899	0.0983	0.0451	0.1949	0.2447
Aluminum alloy 7075-T6	0.9778	2.0462	0.0852	0.0704	0.3041	0.3818
Titanium alloy Ti-6Al-4V	0.7048	1.915	0.0911	0.0512	0.2199	0.2763

**Table 8:** Calculated Poisson’s ratios (ν) for chiral, anti-chiral, and re-entrant structures under X-direction (Case A) and Z-direction (Case B) loading.

Poisson’s Ratio (-)	Chiral Structure		Anti-Chiral Structure		Re-Entrant Structure	
	Case A	Case B	Case A	Case B	Case A	Case B
Structural steel S235	0.2661	−0.4592	−0.7245	−1.2419	−1.9838	−1.0847
Copper alloy C110	0.2676	−0.4588	−0.7244	−1.2555	−1.978	−1.5189
Aluminum alloy 7075-T6	0.2678	−0.4263	−0.7243	−1.2561	−1.797	−2.0927
Titanium alloy Ti-6Al-4V	0.2671	−0.5062	−0.7242	−1.2565	−1.759	−2.1717

## Data Availability

The original contributions presented in this study are included in the article. Further inquiries can be directed to the corresponding author.

## References

[1] Kelkar P.U., Kim H.S., Cho K.-H., Kwak J.Y., Kang C.-Y., Song H.-C. (2020). Cellular Auxetic Structures for Mechanical Metamaterials: A Review. Sensors.

[2] Zhou C., Li Q., Sun X., Xiao Z., Yuan H. (2024). Two-Dimensional Pentamode Metamaterials: Properties, Manufacturing, and Applications. Crystals.

[3] Lakes R. (1987). Foam Structures with a Negative Poisson’s Ratio. Science.

[4] Wojciechowski K.W. (2003). Non-chiral, molecular model of negative Poisson ratio in two dimensions. J. Phys. A Math..

[5] Doroszko M., Falkowska A., Seweryn A. (2021). Image-Based Numerical Modeling of the Tensile Deformation Behavior and Mechanical Properties of Additive Manufactured Ti–6Al–4V Diamond Lattice Structures. Mater. Sci. Eng. A.

[6] Yuan S., Song B., Liu G., Yang B., Dai M., Gao Z., Cao S., Zhao M. (2025). Enhanced Compressive Mechanical Properties of Bio-Inspired Lattice Metamaterials with Taper Struts. Materials.

[7] Zhang J., Meng S., Wang B., Xu Y., Shi G., Zhou X. (2024). Bio-Inspired Sinusoidal Metamaterials: Design, 4D Printing, Energy-Absorbing Properties. Machines.

[8] Falkowska A., Doroszko M., Ostapiuk M., Zasińska K., Seweryn A. (2025). Properties of Gyroid Structure of Cobalt-Based Alloy Obtained by LPBF Method. Int. J. Mech. Sci..

[9] Rodriguez N., Ruelas S., Forien J.-B., Dudukovic N., DeOtte J., Rodriguez J., Moran B., Lewicki J.P., Duoss E.B., Oakdale J.S. (2021). 3D Printing of High Viscosity Reinforced Silicone Elastomers. Polymers.

[10] Zhao H., Zhang E., Lu G. (2024). Study on the Equivalent Stiffness of a Local Resonance Metamaterial Concrete Unit Cell. Buildings.

[11] Tabacu S., Badea A., Sandu A. (2023). Complex Analysis of an Auxetic Structure under Compressive Loads. Sustainability.

[12] Li F., Liu S., Ma S., Zhang X. (2024). Optimization of the Thickness Ratio and Roll-Bonding Parameters of Bimetallic Ti/Al Rod for Bending-Dominated Negative Thermal Expansion Metamaterials. Materials.

[13] Sivák P., Frankovský P., Delyová I., Bocko J., Kostka J., Schürger B. (2020). Influence of Different Strain Hardening Models on the Behavior of Materials in the Elastic–Plastic Regime under Cyclic Loading. Materials.

[14] Burrow G.M., Gaylord T.K. (2011). Multi-Beam Interference Advances and Applications: Nano-Electronics, Photonic Crystals, Metamaterials, Subwavelength Structures, Optical Trapping, and Biomedical Structures. Micromachines.

[15] Rathod V.T. (2020). A Review of Acoustic Impedance Matching Techniques for Piezoelectric Sensors and Transducers. Sensors.

[16] Lim T.-C. (2017). Analogies across auxetic models based on deformation mechanism. Phys. Status Solidi RRL.

[17] Chen K., Li K., Wang Y., Zhang Z., Shi Y., Song A., Zhang Y. (2024). Graphene-Tuned, Tightly Coupled Hybrid Plasmonic Meta-Atoms. Nanomaterials.

[18] Delyová I., Frankovský P., Bocko J., Trebuňa P., Živčák J., Schürger B., Janigová S. (2021). Sizing and Topology Optimization of Trusses Using Genetic Algorithm. Materials.

[19] Na B.-W., Zhang H.-N., Fan Y., Wang Y. (2024). A Molecular Dynamics Study on Auxetic Behaviors of Origami Graphene/Cu Nanocomposites. J. Compos. Sci..

[20] Ghavidelnia N., Bodaghi M., Hedayati R. (2021). Idealized 3D Auxetic Mechanical Metamaterial: An Analytical, Numerical, and Experimental Study. Materials.

[21] Bura E., Grodzki W., Seweryn A. (2024). The Experimental and Numerical Investigation of Fracture Behaviour in PMMA Notched Specimens under Biaxial Loading Conditions—Tension with Torsion. Eng. Fract. Mech..

[22] Zhang X., Yang D. (2016). Mechanical Properties of Auxetic Cellular Material Consisting of Re-Entrant Hexagonal Honeycombs. Materials.

[23] Bilski M., Pigłowski P.M., Wojciechowski K.W. (2021). Extreme Poisson’s Ratios of Honeycomb, Re-Entrant, and Zig-Zag Crystals of Binary Hard Discs. Symmetry.

[24] Ren X., Das R., Tran P., Ngo T.D., Xie Y.M. (2018). Auxetic metamaterials and structures: A review. Smart Mater. Struct..

[25] Széles L., Horváth R., Cveticanin L. (2024). Analysis of Mechanical Properties and Parameter Dependency of Novel, Doubly Re-Entrant Auxetic Honeycomb Structures. Polymers.

[26] Liu S.-C., Chen Y.-L. (2025). Free Vibration Behavior of CFRP Composite Sandwich Open Circular Cylindrical Shells with 3D Reentrant Negative Poisson’s Ratio Core. Polymers.

[27] Top N., Şahin İ., Gökçe H. (2023). The Mechanical Properties of Functionally Graded Lattice Structures Derived Using Computer-Aided Design for Additive Manufacturing. Appl. Sci..

[28] Wang Z., Chen G., Cao X., Chen W., Li C.B., Li X. (2023). Study on the Effect of Nodal Configuration on the Mechanical Properties of Hexa-Ligamentous Chiral Honeycombs. J. Mar. Sci. Eng..

[29] Franzosi P., Colamartino I., Giustina A., Anghileri M., Boniardi M. (2024). Crashworthiness of Additively Manufactured Auxetic Lattices: Repeated Impacts and Penetration Resistance. Materials.

[30] Plewa J., Płońska M., Feliksik K., Junak G. (2025). Geometric Analysis and Experimental Studies of Hexachiral Structures. Materials.

[31] Gibson L.J., Ashby M.F. (1982). Cellular Solids: Structure and Properties.

[32] Masters I.G., Evans K.E. (1996). Models for the Elastic Deformation of Honeycombs. Compos. Struct..

[33] Yang L., Harrysson O.L.A., West H.A., Cormier D. (2015). Mechanical Properties of 3D Re-entrant Honeycomb Auxetic Structures Realized via Additive Manufacturing. Int. J. Solids Struct..

[34] Zhang W., Ding H., Yang X., Lu Z., Wu W. (2019). In-Plane Mechanical Behavior of a New Star-Re-Entrant Honeycomb. Polymers.

[35] Tashkinov M.A., Shuklinov A.V., Glushkov S.A. (2023). Composites with Re-Entrant Lattice: Effect of Filler on Mechanical Response. Polymers.

[36] Zhang X., Xie S., Jiang Y., Yan C., Liu M. (2023). Crashworthiness of Additively Manufactured Re-Entrant Lattices under Dynamic Loading. Materials.

[37] Zhang W., Qi T., Wang H., Chen X., Li X., Shao J. (2025). The Resistance of X-Shaped Re-Entrant Auxetic Sandwich Beams to Localized Impulsive Loading. Crystals.

[38] Plewa J., Kaczmarek M., Żur K., Wierzbicki Ł. (2024). Mechanical Performance of Re-Entrant Scaffolds Fabricated via Additive Manufacturing for Biomedical Applications. Materials.

[39] Zhou Y., Gao Y., Guo H., Chen L. (2023). Additive Manufacturing of Complex Re-Entrant Honeycombs: Mechanical Behavior and Design Guidelines. Materials.

[40] Qian C., Zhao H., Xu L., Li P. (2022). Finite Element Optimization of Re-Entrant Honeycomb Geometries under Uniaxial Loading. Polymers.

[41] Wu H., Liu Y., Zhao H. (2021). Multi-Material 3D Printed Re-Entrant Lattices with Hybrid Compositions. Polymers.

[42] Tang Y., Li Y., Lu Z., Hu Y. (2023). Gradient Re-Entrant Lattices with Spatially Varying Stiffness for Tailored Deformation. Polymers.

[43] Wojciechowski K.W. (1987). Constant thermodynamic tension Monte Carlo studies of elastic properties of a two-dimensional system of hard cyclic hexamers. Mol. Phys..

[44] Prall D., Lakes R.S. (1997). Properties of a chiral honeycomb with a Poisson’s ratio of—1. Int. J. Mech. Sci..

[45] Brańka A.C., Heyes D.M., Wojciechowski K.W. (2011). Auxeticity of cubic materials under pressure. Phys. Status Solidi B.

[46] Zhou Y., Lu Z., Tang Y. (2022). Deformation Mechanisms of Tri-, Tetra-, and Hexa-Chiral Lattices under Uniaxial Loading. Polymers.

[47] Xu H., Li J., Zhou X. (2022). Design of Morphing Wing Structures Using Auxetic Chiral Lattices. Materials.

[48] Zhang J., Li X., Sun Y. (2023). Tunable Acoustic and Vibration Control with Chiral Auxetic Honeycombs. Appl. Sci..

[49] Chen Y., Wang L., Li J. (2021). Influence of Ligament Thickness and Nodal Connectivity on Auxetic Efficiency of Chiral Lattices. Polymers.

[50] Grima J.N., Gatt R., Farrugia P.-S. (2008). On the Properties of Auxetic Meta-Tetrachiral Structures. Phys. Status Solidi B.

[51] Ebrahimi H., Mousanezhad D., Ghosh R., Hamouda A.M.S., Nayeb-Hashemi H., Vaziri A. (2018). 3D cellular metamaterials with planar anti-chiral topology. Mater. Des..

[52] Fleisch J., Khare K., Rimoli J. (2022). Energy absorption in chiral and anti-chiral mechanical metamaterials. Appl. Phys. Lett..

[53] Kalogeropoulou M. (2024). Blueprints of Architected Materials: A Guide to Metamaterial Design for Tissue Engineering. Adv. Mater..

[54] Chen Y., Hu G., Huang Z. (2013). Elasticity of anti-tetrachiral anisotropic lattices. Int. J. Mech. Sci..

[55] Mrozek A., Strek T. (2022). Numerical Analysis of Dynamic Properties of an Auxetic Structure with Rotating Squares with Holes. Materials.

[56] Jiang Y., Liu L., Zhang Y., Zhang H. (2023). Design and Additive Manufacturing of Re-Entrant Lattice Structures with Tailored Mechanical Properties. Polymers.

[57] Xue Y., Li Y., Zhao X., Wang H. (2024). Finite Element Investigation of Chiral Lattices: Influence of Node Rotation and Loading Direction on Auxetic Response. Materials.

[58] Zhang J., Wu C., Wang Y. (2023). Numerical and Experimental Study of Anti-Chiral Auxetic Structures with Isotropic Mechanical Response. Appl. Sci..

